# Slowdown in mortality improvements and trends in lifespan inequality across high-income countries: the role of changing causes of death, 2010–2021

**DOI:** 10.1093/eurpub/ckag002

**Published:** 2026-02-03

**Authors:** Yan Zheng, Alyson van Raalte, Isaac Sasson

**Affiliations:** Department of Sociology and Anthropology, Tel Aviv University, Tel Aviv, Israel; Max Planck Institute for Demographic Research, Rostock, Germany; Max Planck – University of Helsinki Center for Social Inequalities in Population Health (MaxHel Center), Helsinki, Finland; Department of Sociology and Anthropology, Tel Aviv University, Tel Aviv, Israel; Herczeg Institute on Aging, Tel Aviv University, Tel Aviv, Israel

## Abstract

**Background:**

In recent years, several high-income countries have experienced slowdowns in mortality improvements. Prior research has focused on how those changes have impacted life expectancy, but little is known about how they affected lifespan inequality.

**Methods:**

Using data from the Human Mortality Database and World Health Organization Mortality Database, we quantify the contributions of changes in age- and cause-specific mortality to changes in lifespan inequality, measured by life disparity, across 20 high-income countries between 2010 and 2021.

**Results:**

On average, life disparity declined by 0.3 years among both females and males between 2010 and 2019, with declines observed in all countries except for Canada and the United States. These declines were largely attributed to reductions in premature mortality, particularly from cardiovascular diseases and malignant neoplasms. Across English-speaking countries, these reductions were partly (e.g. Canada) or entirely (USA) offset by rising premature mortality related to neuropsychiatric conditions—mainly drug and alcohol use disorders. The COVID-19 pandemic was marked by increasing premature and old-age mortality, which partly contributed to the declines in life disparity in several, though not all, countries.

**Conclusion:**

Mortality dynamics since the 2010s have resulted in distinct changes in lifespan inequality, in addition to life expectancy, across high-income countries. Despite the slowdowns in mortality improvements, lifespans in most countries have become more equal in the decade preceding COVID-19. This trend was interrupted or reversed by the pandemic in many countries. Canada and the United Sates stood out in both periods due to rising premature mortality related to drug and alcohol use.

## Introduction

The second half of the twentieth century was marked by tremendous improvements in mortality and life expectancy across high-income countries. However, some of those countries have experienced slowdowns or reversals in this process during the second decade of the twenty-first century [1, 2]. Underlying these changes were stagnation and even an increase in mortality from several causes of death [2]. The long-standing decline in cardiovascular disease (CVD) mortality, observed since the 1970s, has decelerated in recent years among many high-income countries [3, 4]. At the same time, premature mortality from external causes, especially those associated with ‘deaths of despair’, has become a major health concern in several countries, beginning with the United States [5]. The sudden emergence of COVID-19 has also had a profound adverse impact on global mortality and life expectancy, including most high-income countries [6, 7]. Even prior to the COVID-19 pandemic, rising mortality from influenza and pneumonia in 2014–15 led to periodic reductions in life expectancy in several high-income countries [8].

Life expectancy at birth is a useful summary measure of mortality and population health. However, it can mask considerable heterogeneity in survival patterns across individuals [9]. Lifespan inequality has therefore been proposed as a complementary measure of the spread or variability in ages at death [10]. Although life expectancy and lifespan inequality are negatively correlated—as life expectancy increased historically, lifespans tended to become more equal—this correlation appears to weaken as life expectancy at birth surpasses 70 years [11]. The correlation has also weakened during periods of mortality crises, whether driven by sociopolitical upheavals, famines, or epidemics [12, 13]. Therefore, it is important to examine trends in lifespan inequality and how they relate to underlying age- and cause-specific mortality dynamics. Whereas previous studies examined the impact of slowdowns in mortality improvements on life expectancy across high-income countries [1, 2], their impacts on lifespan inequality remain unknown.

The level of lifespan inequality in society bears critical implications for public health from both individual and societal standpoints [10]. Rising lifespan inequality may indicate worsening population health or emerging public health risks [10, 14]. This study aims to improve our understanding of how and why lifespan inequality has changed across high-income countries between 2010 and 2021, a period marked by slowdowns in mortality improvements and the mortality shock of the COVID-19 pandemic. The findings of this study illuminate how these changes in mortality, representing a break from the long-term trend that preceded them, have impacted the distribution of lifespans within societies, in addition to their average levels.

## Methods

Our analysis includes 20 high-income countries in which high-quality mortality data were available: Australia, Belgium, Bulgaria, Canada, Czechia, Denmark, Finland, France, Israel, Italy, Japan, Lithuania, the Netherlands, Poland, South Korea, Spain, Sweden, Switzerland, the UK, and the USA. For these countries, we obtained age-specific mortality rates by sex between 2010 and 2021 from the Human Mortality Database (HMD) [15], which has set the standard for demographic research on mortality and has been widely used for cross-national comparisons of life expectancy and lifespan inequality [16]. Since the HMD data series for Israel ended in 2016, comparable data were obtained from the Israel Central Bureau of Statistics, to which we applied the HMD protocol [17].

Data on the principal causes of death by age and sex were obtained from the World Health Organization (WHO) Mortality Database [18], a compilation of mortality data based on civil registration and vital statistics in member countries. In this study, we considered eight major cause-of-death groups, namely COVID-19, respiratory infections (including lower and upper respiratory infections), malignant neoplasms, CVD, neuropsychiatric conditions (including alcohol and drug use disorders, which consisted on avreage of over 50% of deaths below age 65 among males and over 30% among females in this category), chronic respiratory diseases (including chronic obstructive pulmonary disease and asthma), unintentional injuries (including road traffic accidents), and others (ICD-10 codes are listed in Table S1). COVID-19 was separated from respiratory infections as a single category. Ill-defined diseases were proportionally redistributed across cause-of-death groups, as proposed by prior research [19]. In the WHO Mortality Database, cause-related mortality data are provided for 5-year aggregate age groups. In order to estimate cause-specific contributions to lifespan inequality, we assumed that the proportion of deaths by cause in the WHO Mortality Database remains constant within each age interval and then applied those proportions to single years of age deaths and exposures in the HMD.

Lifespan inequality can be measured by both absolute indicators (e.g. standard deviation and life disparity) and relative indicators (e.g. coefficient of variation and lifetable entropy) [10, 20]. While they are highly correlated with one another, absolute indicators are generally more easily interpretable because they are expressed in terms of years [13]. In this study, we used life disparity, an absolute indicator defined as the average number of years lost attributable to death [16, 21], to measure lifespan inequality. It can be expressed as


$$e^{†}=\int_{0}^{\omega }d\left(x\right)e\left(x\right)dx,$$

where ω is the highest age of the population, d(x) is the distribution of deaths, and e(x) is life expectancy at age x.

Life disparity is additively decomposable into contributions from ‘early’ and ‘late’ deaths, separated by a threshold age [22], which is especially appealing from a public health perspective [12, 23]. Reducing mortality at ‘early’ ages would lower life disparity by compressing the lower end of the age-at-death distribution toward the threshold, whereas reducing mortality at ‘late’ ages would increase life disparity by expanding the age-at-death distribution away from the threshold. Thus, rising levels of life disparity can result from both favorable and unfavorable changes in mortality. For this reason, we conducted a detailed analysis of the change in life disparity by age-specific contributions. To evaluate the contributions of ‘early’ and ‘late’ deaths to total life disparity change, we used the average threshold age for each sex and each country over the period (Fig. S1).

We applied the Horiuchi decomposition method [24] using the R-package DemoDecomp [25] to decompose the changes in life disparity by sex in each country. Age- and cause-specific contributions were aggregated into two categories: (i) the ‘early’ component, comprising contributions made below the threshold age, and (ii) the ‘late’ component, comprising contributions made above the threshold age. We divided the study period into 2, in order to distinguish between changes in lifespan inequality during the decade preceding the COVID-19 pandemic (2010–19) and the immediate impact of the pandemic (2019–21) across countries. While the two periods differ in length, our objective was not to compare the pace of change between them, but rather to examine whether the pandemic disrupted preexisting patterns across countries.

## Results

### Trends in life disparity

Table 1 shows the change in life disparity across countries. On average, life disparity was estimated at 9.4 years in 2010, 9.1 years in 2019, and 9.3 years in 2021 for females; and 10.9 years, 10.6 years, and 10.6 years for males, respectively. Between 2010 and 2019, the largest decline in life disparity was observed for females in South Korea (−0.7 years), followed by Denmark, Israel, and Lithuania (−0.6 years). Among males, the largest reduction was observed in Lithuania (−1.1 years), followed by Finland and South Korea (−0.7 years). Increases in life disparity were observed only in the USA (0.1 years for females and 0.4 years for males) and Canada (0.1 years for males).

**Table 1.** Life disparity in selected high-income countries, 2010, 2019, and 2021, and change over time, females and males

	Females			Males		
Country	2010	2010–19	2019–21	2010	2010–19	2019–21
Australia	9.1	−0.2	0.0	10.3	0.0	−0.1
Belgium	9.4	−0.3	0.2	10.8	−0.4	0.1
Bulgaria	10.1	−0.2	0.6	12.3	−0.3	−0.4
Canada	9.8	−0.1	0.3	10.7	0.1	0.6
Czechia	9.2	−0.1	0.2	11.0	−0.3	−0.1
Denmark	9.8	−0.6	−0.1	10.6	−0.6	−0.2
Finland	9.1	−0.2	−0.1	11.2	−0.7	−0.1
France	9.4	−0.2	0.0	11.3	−0.4	0.0
Israel	9.2	−0.6	0.0	10.7	−0.6	0.0
Italy	8.7	−0.2	0.1	9.9	−0.4	0.2
Japan	9.0	−0.4	0.0	10.3	−0.4	0.0
Lithuania	10.5	−0.6	0.2	13.9	−1.1	−0.3
Netherlands	9.3	−0.3	0.1	9.8	−0.3	0.0
Poland	9.8	−0.1	0.1	12.4	−0.2	−0.3
South Korea	9.0	−0.7	0.0	10.8	−0.7	−0.1
Spain	8.6	−0.2	0.2	10.4	−0.4	0.1
Sweden	8.9	−0.1	0.0	9.7	−0.3	0.1
Switzerland	8.8	−0.3	0.0	9.8	−0.3	0.1
UK	9.7	−0.2	0.2	10.6	−0.1	0.3
USA	10.8	0.1	0.8	12.1	0.4	0.9

In 2019–21, declines in life disparity continued in a number of countries, mainly among males. The largest reduction in male life disparity was in Bulgaria (−0.4 years), followed by Lithuania and Poland (−0.3 years). In contrast, several countries experienced increases in life disparity, with the USA exhibiting the most notable increases, both among females (0.8 years) and males (0.9 years). Life disparity also increased among females in Bulgaria and among males in Canada by 0.6 years. In other countries, including Australia, Israel, Japan, and South Korea, no notable change in life disparity was observed in either female or male life disparity.

### The contribution of ‘early’ and ‘late’ deaths to the change in life disparity

**Figure 1.**
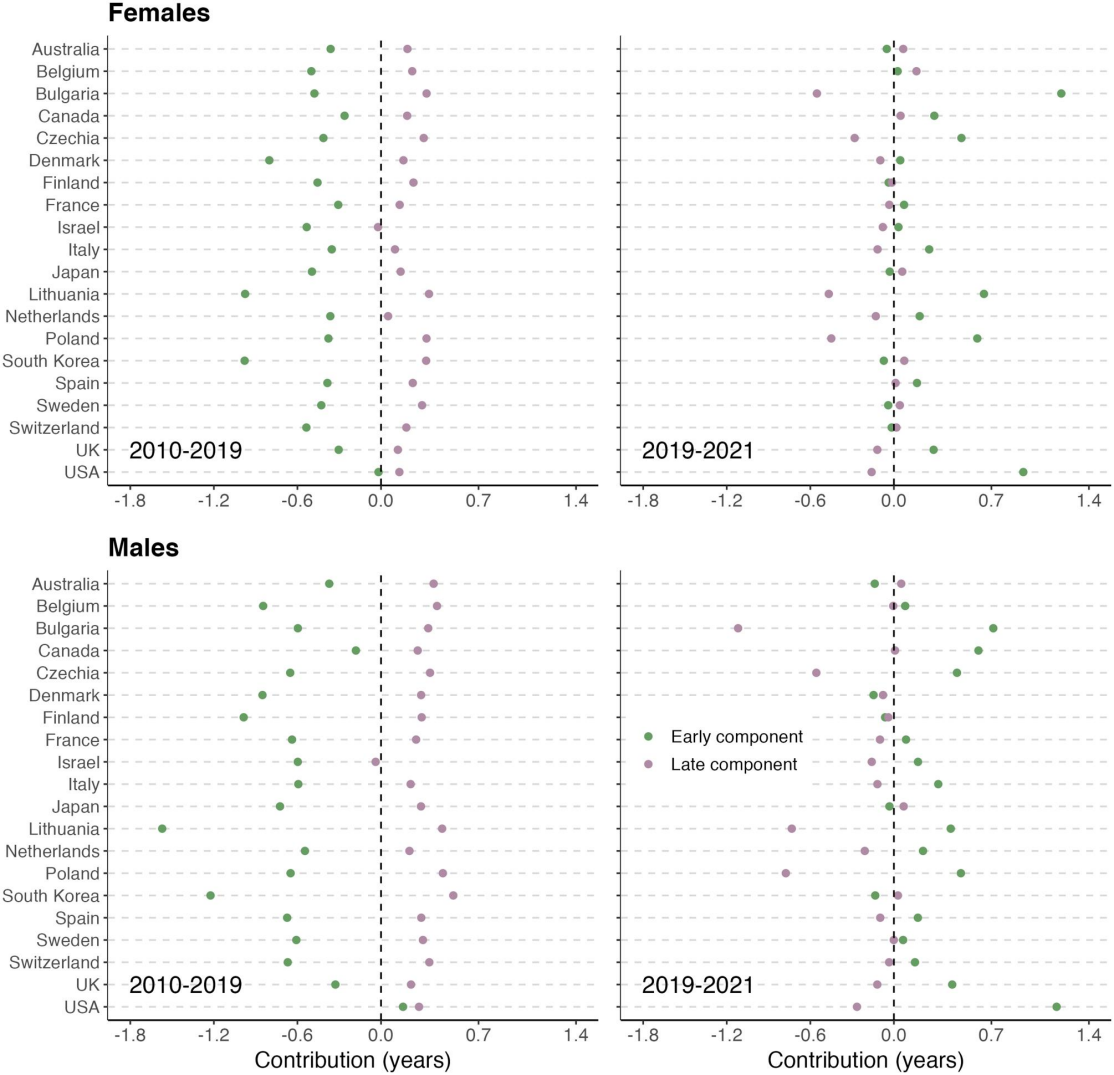
Age-specific contributions to change in life disparity across selected high-income countries for females and males, 2010–19 and 2019–21.

Figure 1 illustrates the contribution of changes in age-specific mortality to changes in life disparity. Between 2010 and 2019, contributions from ‘early’ deaths tended to overshadow contributions from ‘late’ deaths, resulting in net declines in life disparity in most countries. However, in the USA, the contribution to life disparity from ‘early’ deaths was negligible among females, resulting in a modest increase in life disparity attributed to mortality reductions at very old ages (the ‘late’ component). Among males, both components contributed to the increase in life disparity, explained by rising premature mortality, on the one hand, and a reduction in old-age mortality, on the other hand. In contrast, the ‘late’ component had a negligible contribution to changes in life disparity in Israel (both sexes) and the Netherlands (females). In those cases, declines in life disparity were almost exclusively attributed to reductions in premature mortality.

In 2019–21, declines in life disparity were observed mainly among males in Bulgaria, Lithuania, and Poland. However, the primary contributor to those reductions was the impact of rising old-age mortality. In contrast, increases in life disparity in countries such as Bulgaria (females), Canada (males), and the USA (both sexes) were due to the smaller impacts of the ‘late’ component relative to the impacts of the ‘early’ component (i.e. rising premature mortality). In several other countries, the ‘early’ and ‘late’ components offset one another, resulting in no discernible change in life disparity.

### Cause-specific contributions to change in life disparity

**Figure 2.**
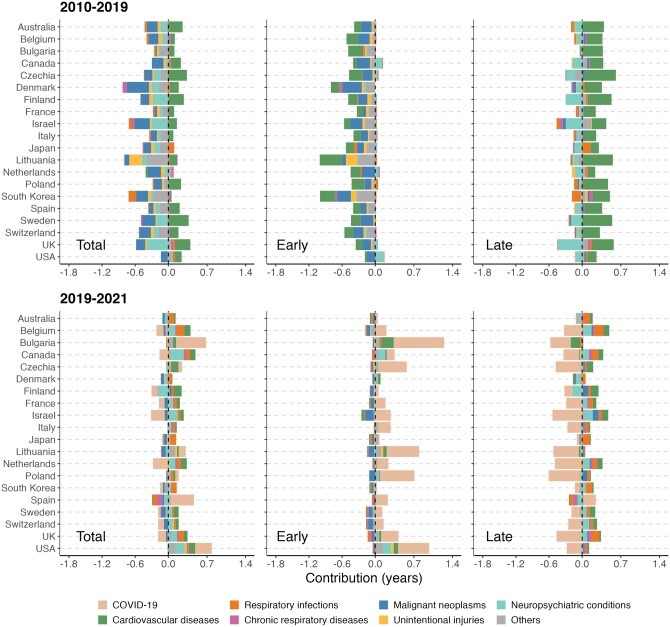
Cause-specific contributions to change in life disparity (total, early, and late components) across selected high-income countries, females, 2010–19 and 2019–21.

**Figure 3.**
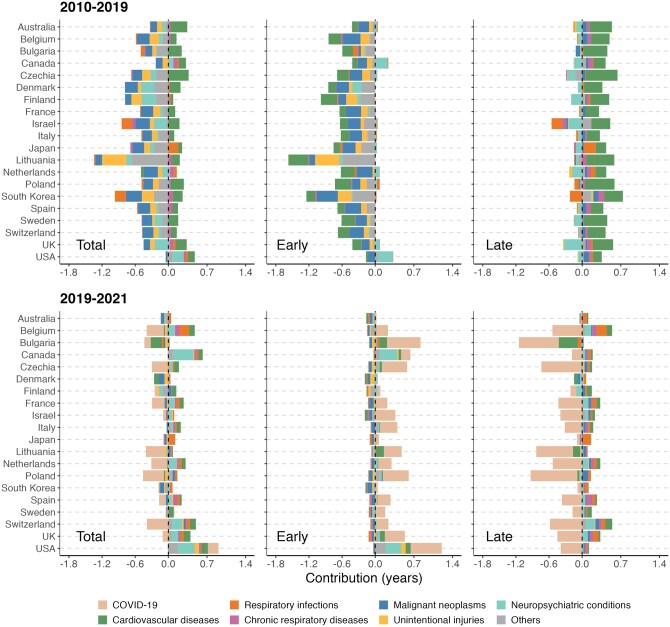
Cause-specific contributions to change in life disparity (total, early and late components) across selected high-income countries, males, 2010–19 and 2019–21.

Figures 2 and 3 show the cause-specific contributions to life disparity changes. In 2010–19, reductions in premature mortality from CVD and malignant neoplasms had large impacts on ‘early’ deaths in nearly all countries included in the study. It is worth noting that considerable reductions in CVD mortality also occurred at older ages (‘late’ deaths), which hindered further declines in life disparity. Reductions in mortality from unintentional injuries at younger ages also contributed to declines in life disparity, but the impact was more notable among males and in countries such as Lithuania and South Korea. In most countries, mortality from neuropsychiatric conditions increased at older ages, contributing to reductions in overall life disparity. However, in Canada and the USA, premature mortality from neuropsychiatric conditions increased as well during this decade, thereby contributing to rising life disparity. In countries such as Israel and South Korea, rising old-age mortality from respiratory infections contributed to reductions in life disparity, particularly among males.

During the period 2019–21, COVID-19 played a primary role in driving changes in life disparity among males and females alike. In many countries, the pandemic raised old-age mortality (the ‘late’ component) more than it had premature mortality (the ‘early’ component), thus reducing life disparity overall. Declines in old-age mortality from infectious respiratory diseases were also observed in many countries, which partly mitigated the COVID-19 effect on life disparity. In Bulgaria, apart from the pandemic factor, the decline in male life disparity was also related to the increase in mortality from CVD in older age groups. In the USA and Canada, the increases in male life disparity were mainly attributed to the rising premature mortality from COVID-19, neuropsychiatric conditions, and other causes.

## Discussion

Despite the slowdown in mortality improvements since the 2010s, most high-income countries included in the study—with the exception of Canada and the United States—continued to show declines in lifespan inequality. However, this trend did not persist in the aftermath of the COVID-19 pandemic, during which declines in lifespan inequality halted and even reversed in multiple countries as a result of changes in underlying age-specific mortality patterns. Even prior to the COVID-19 pandemic, considerable changes in cause-specific mortality were found across high-income countries and have left their mark on lifespan inequality.

### Cardiovascular diseases

In comparison with other causes of death, declines in CVD mortality in recent decades have been more uniformly distributed across age groups (i.e. both below and over the threshold age). Thus, despite the overall positive contribution to life expectancy, the impact of declining CVD mortality on lifespan inequality was rather limited—reductions in CVD mortality tended to compress lifespans below the threshold age upward and at the same time expand the death distribution above the threshold. This general pattern has also been reported in prior research [26]. Recent evidence, however, has indicated slowdowns in the pace of declines in CVD mortality in several high-income countries, particularly among individuals under the age of 75 [3]. The present study finds that during the decade 2010–19, this uneven progress in CVD mortality has contributed to rising lifespan inequality, net of changes from other causes, in nearly all of the observed countries. In other words, declines in CVD mortality at older ages outpaced those at younger ages, thereby making lifespans less equal over time.

This dynamic was more pronounced in the USA, where little to no progress in CVD mortality was observed below the threshold age among males and females alike [27]. In Central and Eastern European countries, CVD mortality has played an important role in shaping lifespan inequality in the mid- to late 1990s and the first decade of the twenty-first century [12]. Our study finds that declining CVD mortality continued to be a major driver of changes in life disparity in those countries in the 2010s. During the COVID-19 pandemic, CVD mortality increased among both middle-aged and older adults in Bulgaria and Lithuania, possibly due to reduced access to healthcare services or policy measures taken during the pandemic [28]. However, since CVD mortality increased below and above the threshold age, it did not affect the overall life disparity in Lithuania. These findings may also underscore persistent gaps in CVD mortality between Central and Eastern European countries relative to Western and Southern European counterparts—a disadvantage that could be associated with higher prevalence of several risk factors, including tobacco use and alcohol consumption [29, 30].

### Neuropsychiatric conditions

Premature mortality associated with neuropsychiatric conditions has increased in several countries during the study period, most notably Canada and the USA, contributing to rising life disparity. However, this category includes diverse causes of death, which vary considerably by age. At younger ages, deaths in this category are dominated by alcohol and drug use disorders, which have been studied extensively before [8, 31]. Among older individuals, this category is dominated by mortality associated with dementia, which increased in many countries during the first period and contributed to reducing lifespan inequality. However, these results should be interpreted with caution, as the increases in dementia-related mortality in many European countries and in Israel, as well as differences between countries, may be attributed to increased awareness and changes in cause-of-death classification practices [4, 32]. Trends in old-age mortality attributed to neuropsychiatric conditions reversed during the pandemic, possibly because the oldest and most frail individuals in the population were most likely to die from COVID-19.

### COVID-19 and other infectious respiratory diseases

Prior research has estimated the reduction in life expectancy that occurred during the COVID-19 pandemic [6, 7]. However, this impact varied considerably across countries, with several (e.g. Australia, Denmark, Japan, and South Korea) showing no discernible change or only minor declines in life expectancy [6, 7, 33, 34]. The present study set out to examine the impact of the pandemic on lifespan inequality across high-income countries. Unlike life expectancy, the findings suggest that this impact was rather limited. Whereas increasing mortality at any age lowers life expectancy, lifespan inequality indices are more sensitive to the specific age pattern in which mortality increases [16, 22]. In the case of the COVID-19 pandemic, mortality tended to increase disproportionately among males and older adults [6, 7]. It did so, however, to varying degrees across countries, which was partly responsible for the negligible change in the overall lifespan inequalities in some, small declines in others, and large increases in a handful of those countries.

With respect to other infectious respiratory diseases, the increasing mortality in some countries, such as Israel and South Korea, among older adults prior to the pandemic may be related to influenza and pneumonia [32, 35]. During the pandemic, however, old-age mortality from infectious respiratory diseases other than COVID-19 declined in most countries, which may be related to measures taken to curtail the pandemic or the emergence of a competing risk among frail individuals.

### Other notable causes of death

In accordance with prior research, this study found the importance of malignant neoplasms and unintentional injuries in shaping lifespan inequality [12, 26]. In almost all high-income countries examined in this study, declines in mortality from malignant neoplasms mainly occurred at younger ages, thus contributing to the declines in life disparity over the observed period. Moreover, declines in premature mortality from unintentional injuries contributed to the overall reduction in life disparity in some observed countries, particularly among males.

### Implications

Medical advancements in treatment and early diagnosis, coupled with effective public intervention programs, have led to considerable progress in reducing CVD and cancer mortality in recent decades. However, the recent slowdown, and in some countries reversal, of the long-term decline in premature mortality hindered further reductions in lifespan inequality, which pose a challenge for achieving both longer and more equal lifespans. To ensure more equal lifespans in the context of changing mortality patterns and emerging health risks, special attention should be given to those causes that disproportionately affect younger adults. Such efforts would entail specific policy interventions to target potential risk factors, including obesity, poor dietary habits, and smoking [2, 3]. In addition, the recurrence of infectious diseases (particularly respiratory) since the 2010s—including the severe 2014–15 influenza season and the recent COVID-19 pandemic—has not only caused reductions in life expectancy in most high-income countries [6–8], but also substantially impacted lifespan inequality. In order to reduce excess mortality at older ages, it is vital to increase the vaccination coverage and ensure prompt and effective interventions against emerging health risks. Furthermore, given the heterogeneous levels and trends in lifespan inequality across countries, the findings in this study underscore the need for country-specific health policies. For instance, worsening mortality conditions among young and middle-aged Americans is alarming, and it places the USA at a disadvantage relative to other high-income countries in terms of both life expectancy and lifespan inequality. Several Central and Eastern European countries are also lagging behind their Western and Southern European counterparts, which suggests that further efforts are necessary to narrow the gaps and achieve regional health equity.

This study is not without limitations. First, the analysis was conducted at the national level. Yet, lifespan inequality in high-income countries could be affected by population heterogeneity (e.g. different mortality patterns across ethnic, socioeconomic, or regional groupings) [10, 36]. Country-specific analyses may shed further light on how compositional changes and mortality dynamics across subpopulations have shaped the national level of lifespan inequality. Second, the period covered by our study ends in 2021, at the height of the COVID-19 pandemic. We are therefore unable to comment on the longer-term impact of the pandemic on lifespan inequality, nor about why this impact varied across countries. Further research is needed to provide a comprehensive account of how the pandemic impacted lifespan inequality in different countries. Lastly, our analysis was based on the assumption that the cause-specific proportions of deaths remain constant within age intervals, which may result in minor biases in our estimates. As more detailed data become available, nuanced analyses can facilitate a more precise understanding of the contributions of age-specific mortality patterns to changes in lifespan inequality.

Despite the slowdowns in mortality improvements since the 2010s, many high-income countries continued to achieve reductions in lifespan inequality in the second decade of the twenty-first century. However, these reductions came to a halt with the emergence of the COVID-19 pandemic, which reversed the trend in some high-income countries. Recent years have been characterized by repeated mortality shocks and crises, ranging from seasonal influenza among older adults to rising ‘deaths of despair’ among younger individuals. These shocks have affected not only life expectancy, but also how equally lifespans are distributed in the population. Preventing premature mortality from multiple causes of death remains a key challenge in ensuring that longer lives are shared more equally in society.

Key points

- The study investigated changes in lifespan inequality across 20 high-income countries from 2010 to 2021, and their underlying age- and cause-specific mortality dynamics.
- Life disparity decreased in nearly all observed countries between 2010 and 2019, except for Canada (among males) and the United States (both sexes), because reductions in premature mortality outpaced reductions in old-age mortality.
- Increasing premature mortality from neuropsychiatric conditions—primarily alcohol and drug use disorders—has partly contributed to rising lifespan inequality in Canada and the United States.
- The slowdown, and in some countries, the reversal in declining premature mortality, hindered further reductions in lifespan inequality, which posed a challenge for achieving both longer and more equal lifespans.

## Supplementary Material

ckag002_Supplementary_Data

## Data Availability

The data used in the study can be obtained at www.mortality.org and https://platform.who.int/mortality.
