# Supplementary material for: Slowdown in mortality improvements and trends in lifespan inequality across high-income countries: the role of changing causes of death, 2010–2021
Source: Eur J Public Health. 2026 Feb 3;36(2):ckag002. doi: 10.1093/eurpub/ckag002 (PMC13017460; doi:10.1093/eurpub/ckag002)
Supplement: ckag002_Supplementary_Data [file ckag002_supplementary_data.zip › 21-Jan-2026_010231_ejph-2025-03-om-0215-File005.docx]

**Slowdowns in mortality improvements and trends in lifespan inequality across high-income countries: The role of changing causes of death, 2010-2021**

Yan Zheng, Alyson van Raalte & Isaac Sasson

Supplementary material

Table S1. ICD codes included in each cause-of-death category

| **Cause of death** | **ICD codes** |
| --- | --- |
| COVID-19 | U07.1, U07.2, U09.9, U10.9 |
| Respiratory infections (excluding COVID-19) | H65-H66, J00-J22, P23, U04 |
| Malignant neoplasms | C00-C97 |
| Neuropsychiatric conditions | F01-F99, G06-G98 (minus G14), U07.0, X41, X42, X44, X45 |
| Cardiovascular diseases | I00-I99 |
| Chronic respiratory diseases | J30-J98 |
| Unintentional injuries | V01-X59, Y40-Y86, Y88, Y89 (minus X41-X42, X44-X45), U12.9 |
| Ill-defined diseases | R00-R94, R96-R99 |


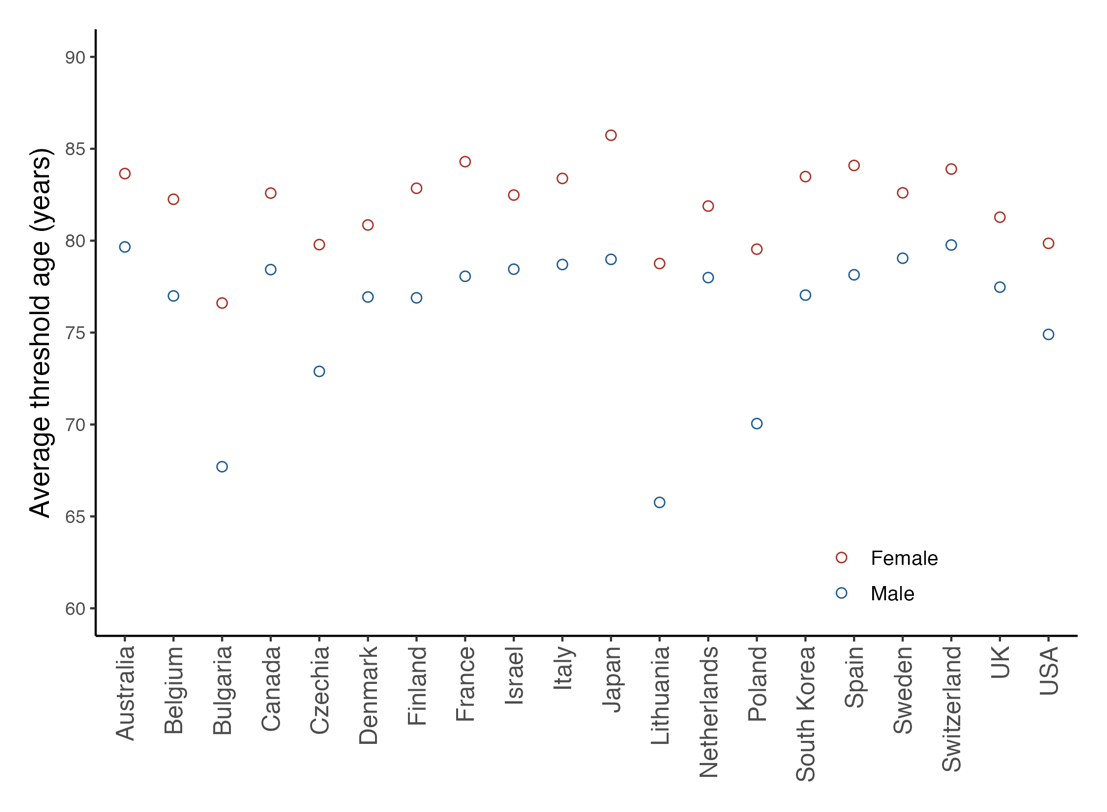


Figure S1. Average threshold age by sex across high-income countries for females and males, 2010-2021
